# Phase I Study of [^68^Ga]Ga-Anti-CD206-sdAb for PET/CT Assessment of Protumorigenic Macrophage Presence in Solid Tumors (MMR Phase I)

**DOI:** 10.2967/jnumed.122.264853

**Published:** 2023-09

**Authors:** Odrade Gondry, Catarina Xavier, Laurens Raes, Johannes Heemskerk, Nick Devoogdt, Hendrik Everaert, Karine Breckpot, Quentin Lecocq, Lore Decoster, Christel Fontaine, Denis Schallier, Sandrine Aspeslagh, Ilse Vaneycken, Geert Raes, Jo A. Van Ginderachter, Tony Lahoutte, Vicky Caveliers, Marleen Keyaerts

**Affiliations:** 1 MIMA, Department of Medical Imaging, Vrije Universiteit Brussel, Brussels, Belgium;; 2 Nuclear Medicine Department, Universitair Ziekenhuis Brussel, Brussels, Belgium;; 3 Laboratory for Molecular and Cellular Therapy, Vrije Universiteit Brussel, Brussels, Belgium;; 4 Department of Medical Oncology, Universitair Ziekenhuis Brussel, Brussels, Belgium;; 5 Cellular and Molecular Immunology, Lab of Cellular and Molecular Immunology, Vrije Universiteit Brussel, Brussels, Belgium; and; 6 Myeloid Cell Immunology Lab, VIB Center for Inflammation Research, Brussels, Belgium

**Keywords:** CD206, single-domain antibody, PET/CT, phase I, tumor-associated macrophages

## Abstract

Macrophages play an important role throughout the body. Antiinflammatory macrophages expressing the macrophage mannose receptor (MMR, CD206) are involved in disease development, ranging from oncology to atherosclerosis and rheumatoid arthritis. [^68^Ga]Ga-NOTA-anti-CD206 single-domain antibody (sdAb) is a PET tracer targeting CD206. This first-in-human study, as its primary objective, evaluated the safety, biodistribution, and dosimetry of this tracer. The secondary objective was to assess its tumor uptake. **Methods:** Seven patients with a solid tumor of at least 10 mm, an Eastern Cooperative Oncology Group score of 0 or 1, and good renal and hepatic function were included. Safety was evaluated using clinical examination and blood sampling before and after injection. For biodistribution and dosimetry, PET/CT was performed at 11, 90, and 150 min after injection; organs showing tracer uptake were delineated, and dosimetry was evaluated. Blood samples were obtained at selected time points for blood clearance. Metabolites in blood and urine were assessed. **Results:** Seven patients were injected with, on average, 191 MBq of [^68^Ga]Ga-NOTA-anti-CD206-sdAb. Only 1 transient adverse event of mild severity was considered to be possibly, although unlikely, related to the study drug (headache, Common Terminology Criteria for Adverse Events grade 1). The blood clearance was fast, with less than 20% of the injected activity remaining after 80 min. There was uptake in the liver, kidneys, spleen, adrenals, and red bone marrow. The average effective dose from the radiopharmaceutical was 4.2 mSv for males and 5.2 mSv for females. No metabolites were detected. Preliminary data of tumor uptake in cancer lesions showed higher uptake in the 3 patients who subsequently progressed than in the 3 patients without progression. One patient could not be evaluated because of technical failure. **Conclusion:** [^68^Ga]Ga-NOTA-anti-CD206-sdAb is safe and well tolerated. It shows rapid blood clearance and renal excretion, enabling high contrast-to-noise imaging at 90 min after injection. The radiation dose is comparable to that of routinely used PET tracers. These findings and the preliminary results in cancer patients warrant further investigation of this tracer in phase II clinical trials.

## Visual Abstract

**Figure:**
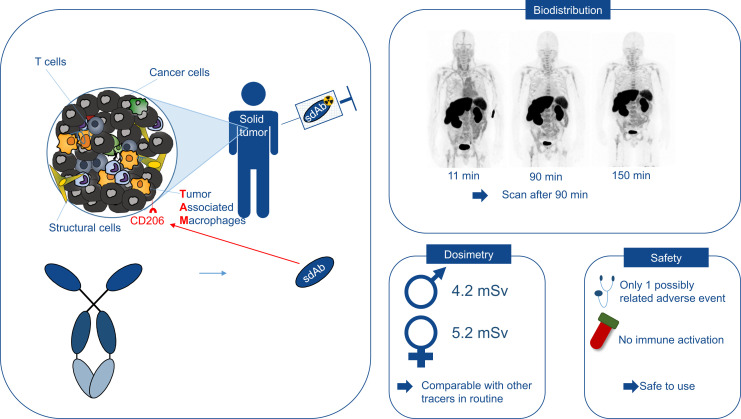

Macrophages are important throughout the body. They play a regulatory role in inflammatory processes and can adopt various activation states. Although a binary M1–M2 macrophage activation paradigm has long been used, we now know that complex macrophage subtypes exist in vivo (1). Macrophages expressing the macrophage mannose receptor (MMR, CD206) are typically considered antiinflammatory.

In oncology, tumor-associated macrophages vary from being proinflammatory or immunostimulatory and antitumoral to being immunosuppressive and protumoral (2–5). Macrophages expressing CD206 play a regulatory role in the angiogenesis, invasion, and migration of cancer cells and are considered protumorigenic (6). Consequently, the presence of CD206-expressing macrophages was shown to correlate with poor responses to multiple types of therapy both in mouse tumor models and in patients (7–9). Beyond oncology, the role of these macrophages is being investigated in many other diseases, such as atherosclerosis, rheumatoid arthritis, sarcoidosis, and inflammatory bowel disease (10–14).

Today, the presence of antiinflammatory macrophages can be assessed by histologic examination of biopsy specimens, limiting the assessment to only a few lesions and small areas within lesions. The development of an anti-CD206 radiotracer would enable noninvasive and whole-body detection of CD206 expression in patients through molecular imaging, allowing different disease sites to be evaluated in a single procedure (15).

Previous efforts in radiotracer development to visualize CD206 expression have mainly focused on the use of mannosylated compounds, because these are naturally binding to CD206. However, mannosylated tracers, such as 2-deoxy-2-[^18^F]fluoro-d-mannose, ^68^Ga-labeled NOTA-neomannosylated human serum albumin, and [^99m^Tc]Tc-Tilmanocept (Lymphoseek; Cardinal Health), can also bind other pathogen-recognition receptors such as CD209, resulting in reduced specificity for the CD206 target (16–19).

Here, we report on a phase I trial to assess this ^68^Ga-labeled, NOTA-coupled, anti-CD206 single-domain antibody (sdAb) for PET/CT imaging, which specifically targets CD206. The primary objective was to assess the safety, biodistribution, and dosimetry. The secondary objective was to evaluate tumor uptake of the radiotracer in patients with malignant solid tumors (20–23).

## MATERIALS AND METHODS

**FIGURE 1.**
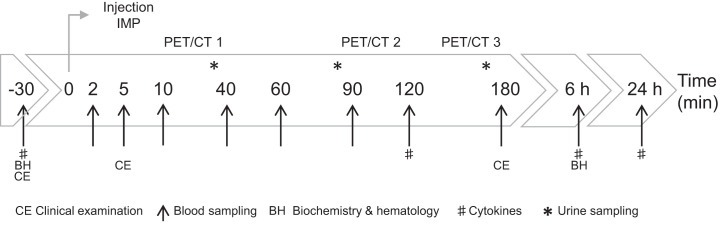
Schematic representation of study procedures over time. IMP = investigational medicinal product.

Full materials and methods can be found in the supplemental materials (supplemental materials are available at http://jnm.snmjournals.org). Figure 1 depicts the trial design.

### Approvals

This single-center, open-label, nonrandomized, first-in-humans clinical trial (EudraCT 2017-001471-23, NCT04168528) evaluated the safety, biodistribution, radiation dosimetry, and tumor imaging potential of [^68^Ga]Ga-NOTA-anti-CD206-sdAb. The study has been approved by the Belgian Federal Agency for Medicines and Health Products, the local ethics committee (Universitair Ziekenhuis Brussel), and the Belgian Federal Agency for Nuclear Control, and all subjects signed an informed consent form. This study is in accordance with the Declaration of Helsinki and the International Conference on Harmonization Guidelines for Good Clinical Practice.

### Patient Selection

Patients with a local, locally advanced, or metastatic solid tumor of at least 10 mm were eligible. Important exclusion criteria were an Eastern Cooperative Oncology Group score higher than 2, estimated glomerular filtration rate below 50 mL/min/1.73 m^2^ (according to the Cockcroft–Gault equation), and abnormal liver function (bilirubin > 1.5 times the upper limit of normal and alanine transaminase > 3 times the upper limit of normal). Supplemental Table 1 shows other inclusion and exclusion criteria.

After a major power outage that occurred shortly after injection of patient 5, PET/CT imaging at preset time points was impossible, resulting in insufficient data. Therefore, the study protocol was amended to include 7 instead of 6 patients. Data from 7 subjects were available for evaluation of safety and tolerability; data from 6 subjects were available for evaluation of biodistribution, dosimetry, and tumor uptake.

### Preparation of the Product

Anti-CD206-sdAb was produced by Q-biologicals NV, now part of Amatsigroup, according to good-manufacturing-practice standards. This patented sdAb is named NbhmMMRm3.49. Its generation is described by Blykers et al. (22). NOTA-anti-CD206-sdAb and [^68^Ga]Ga-NOTA-anti-CD206-sdAb were produced as previously described (23).

### PET/CT Imaging

On average, 191 ± 21 MBq (range, 174–236 MBq) of [^68^Ga]Ga-NOTA-anti-CD206-sdAb, containing an estimated mass of 79.1 ± 14.6 μg (range, 58.2–96.8 μg) of NOTA-anti-CD206-sdAb in 0.9% NaCl containing 5 mg of ascorbic acid (pH 5.9–6.2), was injected as an intravenous bolus. In each patient, 3 PET/CT scans were performed at an average of 11 min (PET/CT 1), 90 min (PET/CT 2), and 150 min (PET/CT 3) after injection.

### Safety Assessment

All patients were interviewed before injection and at 3 and 24 h and thoroughly examined before injection and 3 h after injection. Blood samples to assess hematology, liver enzymes, kidney function, ions, ferritin, and C-reactive protein were obtained before injection and at 6 h after injection. Cytokine analysis was performed on blood samples obtained before injection and at 2, 3, and 24 h after injection.

### Blood and Urine Samples

Peripheral venous blood samples were taken before injection of the compound and at 2, 5, 10, 40, 60, 85, 120, and 180 min after injection. Urine samples were collected at 35 ± 10, 80 ± 10, and 180 ± 10 min after injection. An additional blood sample was collected at least 3 mo after injection and stored, together with a sample before injection, for future antidrug antibody detection (24).

Whole-blood and plasma samples were counted against appropriate standards of known dilution in an automatic γ-well counter and, after correction for decay and background activity, expressed as percentage injected activity. Blood volume was estimated according to body weight and height using the Nadler formula and the patient’s hematocrit (25). Based on the blood samples taken between the time of injection and 3 h after injection, blood and plasma time–activity curves were calculated. Blood half-lives were calculated with a 2-phase exponential decay model using Prism software (GraphPad Software).

### Volume-of-Interest Definition

[^68^Ga]Ga-NOTA-anti-CD206-sdAb uptake in different organs was determined using volumes of interest, drawn with MIM Encore version 7.1.3 (MIM Software Inc.), for the following: blood-pool activity in the left ventricle, liver, spleen, kidneys, adrenals, thyroid, and red bone marrow; urinary activity in the bladder and ureter; and whole-body activity. Biologic half-life was calculated using total-body activity over time, excluding urinary activity.

### Dosimetry

Biodistribution data were expressed as decay-corrected uptake per organ relative to the administered activity. For each organ in every patient, a monoexponential fit was performed in OLINDA/EXM version 1.0 (Vanderbilt University) (26). The total effective dose was calculated for an adult male model and an adult female model.

### SUV Measurements

SUVs were measured using Syngo.via VB40 software (Siemens), with SUVs corrected for body weight. Tracer uptake in the liver, spleen, and bone marrow and left ventricular blood-pool activity were measured by the routinely used SUV_mean_ parameter using a spheroid volume of interest within the organ.

In patients with progressive disease, the largest lesion was measured, as well as lesions that turned out to be progressive and were at least 10 mm. Progression-free survival and overall survival were retrospectively assessed using the available patient data.

## RESULTS

### Patient Characteristics and Inclusion

Seven patients were included. Table 1 summarizes the patient characteristics.

**TABLE 1.** Patient Characteristics

Patient no.	Age (y)	Sex	Cancer type	ECOG score	Current therapy	Previous lines
1	63	Male	NSCLC	0	Pembrolizumab	0
2	69	Male	NSCLC	1	Durvalumab	1
3	64	Male	NSCLC	1	Pembrolizumab	0
4	54	Male	NSCLC	1	Nivolumab	3
5	56	Male	NSCLC	0	Atezolizumab	2
6	66	Male	Atypical carcinoid	1	Watchful waiting	1
7	55	Male	Renal cell carcinoma	1	Nivolumab	3

ECOG = Eastern Cooperative Oncology Group; NSCLC = non–small cell lung carcinoma.

### Safety Assessment

Safety was assessed in 7 subjects. Eight adverse events were documented (Supplemental Table 2). One adverse event (Common Terminology Criteria for Adverse Events grade 1; National Cancer Institute) was possibly related to the radiotracer. It was a transient increase in headache, occurring in a patient with known brain metastases that progressed at follow-up. In 1 patient, a severe adverse event was recorded (stroke, Common Terminology Criteria for Adverse Events grade 3) 56 d after injection and was considered unrelated. Clinical laboratory testing of blood, taken before and 360 min after injection, showed no significant changes that could be related. No increase in blood cytokine levels was observed after injection (Supplemental Fig. 1).

### Pharmacokinetics and Biodistribution

**FIGURE 2.**
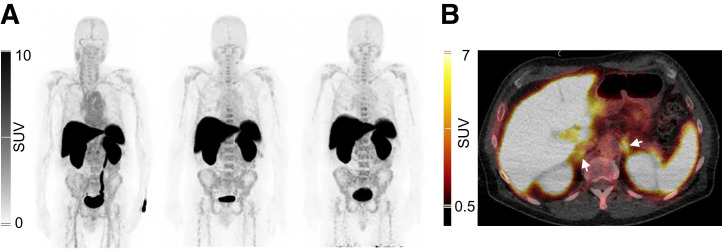
Normal biodistribution of [^68^Ga]Ga-NOTA-anti-CD206-sdAb. (A) Anterior maximum intensity projections of patient 1 at 11 min (left), 90 min (center), and 150 min (right) after injection. (B) Axial PET/CT fusion image at 90 min after injection, showing uptake in liver, spleen, kidneys, and adrenals (arrows). No tracer uptake is visible in lung lesion of this patient.

**FIGURE 3.**
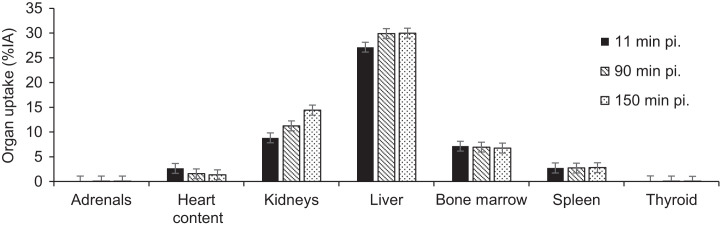
Relative organ uptake over time. Data are decay-corrected to time of injection and shown as mean ± SD in 6 patients. %IA = percentage injected activity; pi = after injection.

Figure 2 shows PET/CT images representative for tracer biodistribution. Blood-pool activity was visible at 11 min after injection, with a clear delineation of the heart and large blood vessels, and subsequently decreases at the later time points. Uptake was mainly seen in the liver, kidneys, spleen, and adrenals, already at 11 min after injection, and remained high at 90 and 150 min after injection. In addition, low uptake in the bone marrow was seen at all time points. Uptake in multiple organs was fast and stable and in the kidneys even increased over time (Fig. 3).

The tracer was renally cleared and excreted in urine. Despite high liver uptake, no signs of hepatobiliary excretion were visible.

SUVs were in line with the measurements presented in Figure 3, confirming the highest average SUV_mean_ of 15.8 ± 2.6 at 90 min after injection for the liver, followed by the spleen and to a lesser extent bone marrow and blood-pool activity measured in the left ventricle (Supplemental Table 3). Only blood-pool activity clearly decreases over time, representing the blood clearance of the tracer, as shown in Supplemental Table 4.

**FIGURE 4.**
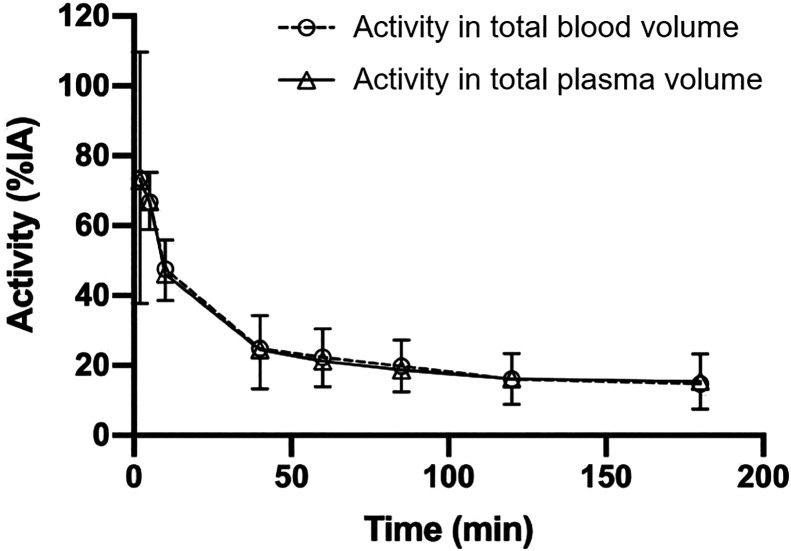
Time–activity curve for total blood volume and total plasma volume, expressed as percentage injected activity (%IA). Data are presented as mean ± SD of 7 patients.

Blood and plasma time–activity curves overlapped, indicating that the tracer is not cell-bound in blood (Fig. 4). Blood and plasma clearance were fast, with only 20% of injected activity remaining in the total blood volume at 85 min after injection (Fig. 4). Blood half-lives were calculated at 7.5 min (early phase) and 78.8 min (late phase).

Biologic half-life was on average 13.9 h (interval, 8.1–19.2 h). This long half-life is explained by retention of the sdAb in the major organs. No metabolites were detected in blood and urine samples.

### Dosimetry

Table 2 summarizes the average specific organ doses and effective dose for 6 subjects. The kidneys showed the highest organ dose (male patients, 0.24 ± 0.05 mGy/MBq; female patients, 0.26 ± 0.06 mGy/MBq), followed by the liver, spleen, and urinary bladder wall.

**TABLE 2.** Organ Doses and Effective Dose

Organ	Male organ dose	Female organ dose
Kidneys	0.241 ± 0.048	0.263 ± 0.0517
Liver	0.135 ± 0.0138	0.181 ± 0.0188
Spleen	0.119 ± 0.0339	0.145 ± 0.0415
Urinary bladder wall	0.0477 ± 0.0118	0.0634 ± 0.0161
Adrenals	0.0475 ± 0.0129	0.0561 ± 0.0150
Thyroid	0.0287 ± 0.0207	0.0346 ± 0.0250
Red bone marrow	0.0267 ± 0.0038	0.0260 ± 0.0035
Heart wall	0.0244 ± 0.0244	0.0288 ± 0.0059
Osteogenic cells	0.0214 ± 0.0025	0.0288 ± 0.0034
Effective dose, ICRP 103	0.0219 ± 0.0010	0.0269 ± 0.0012
Effective dose, ICRP 60	0.0217 ± 0.0012	0.0271 ± 0.0015

ICRP = International Commission on Radiological Protection.

Organ doses are in milligrays per megabecquerel. Effective doses are in millisieverts per megabecquerel.

The average total effective dose (according to International Commission on Radiological Protection publication 103) was 0.02 ± 0.001 mSv/MBq in male patients and 0.03 ± 0.001 mSv/MBq in female patients. Given the mean injected activity of 191 MBq in this study, the average effective dose was 4.2 mSv for male patients and 5.2 mSv for female patients.

### Uptake in Tumor Lesions

**FIGURE 5.**
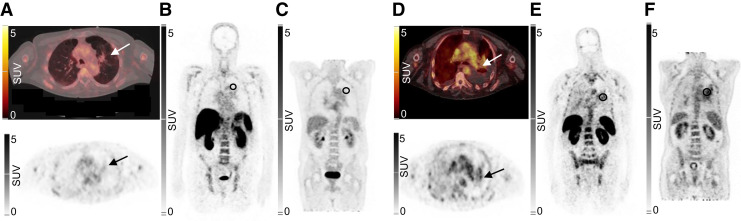
Uptake of [^68^Ga]Ga-NOTA-anti-CD206-sdAb in patient 1 (A–C) and patient 2 (D–F), both with non–small cell lung carcinoma and residual lesion in lung region during treatment. (A and D) Uptake in lung lesion (arrow) on PET/CT (top) and PET (bottom) images 90 min after injection. Coronal PET images show lesional uptake, indicated by ellipsoid in left lung, for [^68^Ga]Ga-NOTA-anti-CD206-sdAb (B) and [^18^F]FDG performed 15 wk after study participation (C) of patient 1, who showed long-term disease remission. Coronal PET images show lesional uptake, indicated by ellipsoid in left lung of patient 2, for [^68^Ga]Ga-NOTA-anti-CD206-sdAb (E) and [^18^F]FDG performed 11 wk later (F). This patient showed disease progression on these [^18^F]FDG PET/CT images.

Uptake in tumor lesions could be evaluated in 6 patients. Tracer uptake above surrounding background was visible in the relevant lesions in 3 of 6 patients, with an SUV_max_ higher than 2.0 g/mL (Table 3). Images showing [^68^Ga]Ga-NOTA-anti-CD206-sdAb uptake in the tumor lesion of patient 2 with an SUV_max_ of 4.0 g/mL are presented in Figures 5D–5F.

**TABLE 3.** SUVs in Selected Lesions

Patient no.	Lesion-of-inclusion location	Injected activity (MBq)	SUV_peak_	SUV_max_	Disease progression after injection*	Time to progression after injection (mo)	Time to death after injection (mo)
1	Left upper lobe	174	1.0	1.2	No	—	Alive*
2	Left upper lobe	184	3.2	4.0	Yes	2	12
3	Mediastinal adenopathy	181	1.0	1.1	No	—	Alive*
4	Left upper lobe	236	NA	2.3	Yes	1	6
6	L3 vertebra		1.8	2.0	Yes	9	20
	Scapula	185	1.8	2.4			
	Subpleural left		2.0	2.4			
7	Left iliac wing	183	0.7	1.0	No	—	Alive*

— = no disease progression; * = patient still alive; NA = not applicable.

Progression is based on PERCIST criteria or, if no [^18^F]FDG PET/CT data were available, on iRECIST criteria (38*,*^39^).

Although this was not part of the prospective study, survival data were retrospectively assessed. Patient 2 showed progression-free survival of 2 mo. For 2 other patients with the highest tumor uptake values (SUV_max_, 2.0 or higher), progression-free survival was 1 mo (patient 4) and 9 mo (patient 6). Three patients with the lowest tumor uptake values all showed progression-free survival until June 2022, which is a minimum of 28 mo after injection. Images of patients 1 and 2 are shown in Figure 5.

## DISCUSSION

[^68^Ga]Ga-NOTA-anti-CD206-sdAb is an sdAb derived from heavy-chain–only antibodies and was previously developed by our research group for imaging of protumorigenic, CD206-expressing macrophages in the tumor microenvironment, as well as in other macrophage-related diseases (10*,*^13^*,*^21^–^23^*,*^27^). We here present the data for safety (*n* = 7) and for biodistribution and dosimetry (*n* = 6) in cancer patients. In addition, we report preliminary tumor uptake results.

For safety, adverse events were monitored using clinical evaluation and blood analysis. In addition, cytokine and ferritin levels were measured in blood, because these can rise in cases of macrophage activation (28–30). No indication of such activation was found, which is in line with the fact that CD206 does not signal intracellularly (30). Only 1 possibly related adverse event was noted, which was of mild severity and, in retrospect, likely resulted from disease progression. Therefore, [^68^Ga]Ga-NOTA-anti-CD206-sdAb is deemed safe for use in future clinical trials.

The biodistribution data are in line with the preclinical results (21*,*^22^): highest tracer uptake is seen in the liver, spleen, and kidneys. Liver uptake likely results from physiologic expression of CD206 by the liver sinusoidal endothelial cells (31). Kidney uptake is explained by the cortical accumulation that is typically seen for peptides and small proteins (32). Uptake in the spleen and bone marrow agrees with the presence of CD206-expressing immune cells in these organs (33).

Fast blood clearance was confirmed for [^68^Ga]Ga-NOTA-anti-CD206-sdAb and will enable imaging at early time points. Because blood level activities did not decrease strongly beyond 90 min, this time point was selected for PET/CT imaging in the ongoing phase II trial. The tracer is cleared by the kidneys, without arguments for additional hepatobiliary clearance. No metabolites were detected in blood and urine samples, indicating the stability of the compound in vivo.

Whole-body and organ dosimetry was performed to assess the radiation burden on the patients. The kidneys received the highest organ dose, which was well below thresholds for deterministic effects. With an average effective dose of 2.2 × 10^−2^ mSv/MBq in the adult male, the expected radiation burden is comparable to that of commonly used tracers such as [^18^F]FDG (1.9 × 10^−2^ mSv/MBq) and [^68^Ga]Ga-PSMA (2.2 × 10^−2^ mSv/MBq) (34*,*^35^).

Other research groups have used mannose-based radiotracers for imaging of CD206-positive macrophages. Tahara et al. reported on 2-deoxy-2-[^18^F]fluoro-d-mannose as a noninferior tracer to [^18^F]FDG for the visualization of high-risk atherosclerotic plaques in a rabbit model (17). ^68^Ga-labeled NOTA-neomannosylated human serum albumin has been investigated in atherosclerosis, myocarditis, and pulmonary artery hypertension in animal models (18*,*^36^*,*^37^). Tilmanocept has been approved for human use, albeit only for local and not for systemic administration, for the indication of sentinel node identification. The data presented here are, to our knowledge, the first human biodistribution data of a CD206-specific radiotracer using systemic administration.

As a secondary and exploratory objective, tumor uptake values of [^68^Ga]Ga-NOTA-anti-CD206-sdAb were quantified and retrospectively confronted with time to progressive disease in these 6 patients. Overall, uptake values were quite low, reaching an SUV_max_ of up to 4.0 g/mL. We observed that 3 of 6 patients, all with an SUV_max_ of 2.0 g/mL or higher, subsequently progressed, whereas the 3 patients with lower tracer uptake had long-term, progression-free survival (Table 3). This preliminary observation on only a small number of subjects needs to be confirmed in prospective clinical trials before conclusions about the predictive potential of [^68^Ga]Ga-NOTA-anti-CD206-sdAb PET in cancer can be made. A phase II trial in which tracer uptake is correlated with CD206 expression using immunohistochemistry is ongoing (NCT04168528). In addition, lesional uptake in patients with different disease types will be assessed to explore the value of [^68^Ga]Ga-NOTA-anti-CD206-sdAb in macrophage-related diseases (NCT04758650). If results would be positive, this tracer could be used in the future to assess the prognostic value of these macrophages for cancer survival and therapy responses, as well as in other inflammatory diseases.

## CONCLUSION

[^68^Ga]Ga-NOTA-anti-CD206-sdAb is a PET tracer that was evaluated in patients to assess its safety, biodistribution, and dosimetry. The tracer was confirmed to be safe, with a radiation burden similar to that of other routinely used PET tracers. It shows uptake in organs with known CD206 expression, such as the liver, spleen, and bone marrow, confirming its specificity. High kidney uptake is observed, as expected for peptides and small proteins. The tracer is cleared by the kidneys. Blood-pool activity decreases quickly over time, enabling high contrast-to-noise imaging at 90 min after injection. Preliminary tumor uptake data in a limited number of patients encourage further evaluation of this tracer in phase II clinical trials.

## DISCLOSURE

This project was funded by VUB-IOF INTEGRAL, Stichting tegen Kanker, and Research Foundation–Flanders. Catarina Xavier, Laurens Raes, Johannes Heemskerk, Hendrik Everaert, Christel Fontaine, Denis Schallier, Ilse Vaneycken, and Vicky Caveliers have nothing to disclose. Odrade Gondry and Quentin Lecocq received an Emmanuel Van der Schueren Grant funded by Kom op tegen Kanker (Stand up to Cancer), the Flemish Cancer Society. Geert Raes, Nick Devoogdt, and Tony Lahoutte are consultants for Precirix. Geert Raes, Nick Devoogdt, Tony Lahoutte, Jo Van Ginderachter, and Marleen Keyaerts are founders and shareholders in Abscint NV/SA. Geert Raes, Nick Devoogdt, Tony Lahoutte, Jo Van Ginderachter, Marleen Keyaerts, Karine Breckpot, and Quentin Lecocq hold patents related to sdAb imaging and therapy. Jo Van Ginderachter, Geert Raes, Nick Devoogdt, Odrade Gondry, Tony Lahoutte, and Marleen Keyaerts are supported by the EU/EFPIA/Innovative Medicines Initiative 2 Joint Undertaking Immune-Image grant 831514. ND reports grants, personal fees, and nonfinancial support from Precirix and nonfinancial support from Abscint. Lore Decoster received fees for advisory boards, speakers, research, and travel grants to Universitair Ziekenhuis Brussel from BMS, MSD, Roche, Bhoeringer Ingelheim, Astra Zeneca, Lilly, and Servier. Sandrine Aspeslagh declares memberships on an advisory board or board of directors for MSD, Sanofi, Roche, BMS, Pfizer, and Galapagos. Tony Lahoutte and Marleen Keyaerts have an FWO clinical mandate. No other potential conflict of interest relevant to this article was reported.

## Notes

KEY POINTS **QUESTION:** What is the safety, biodistribution, and dosimetry of [^68^Ga]Ga-NOTA-anti-CD206-sdAb, a radiopharmaceutical to image protumorigenic macrophages, as well as observations about its tumor-targeting potential in cancer patients? **PERTINENT FINDINGS:** In this monocenter phase I study in 7 cancer patients, tracer safety was confirmed, with an average effective dose of 4.2 mSv. Biodistribution analysis showed tracer uptake in MMR-expressing organs such as the liver, spleen, adrenals, and bone marrow with fast blood clearance, allowing high tissue-to-background imaging at 90 min after injection, and detection of tumor uptake values (SUV_max_) ranging from 1.0 to 4.0. **IMPLICATIONS FOR PATIENT CARE:** Further development of [^68^Ga]Ga-NOTA-anti-CD206-sdAb could enable the quantification of CD206-expressing macrophages in the tumor microenvironment and could help to reveal the role of these cells in local immune-related resistance mechanisms to cancer treatments.
